# Daughters-in-Law in Negotiating the Intergenerational Contract in Rural China: A Qualitative Case Study

**DOI:** 10.1093/geroni/igab046.395

**Published:** 2021-12-17

**Authors:** Yong-Zhen Li, Crystal Kwan

**Affiliations:** The Hong Kong Polytechnic University, Kowloon, Hong Kong

## Abstract

Daughters-in-law play a key role in intergenerational relationships, especially in Rural China. Albeit, their voices are less heard and examined. This study explores how daughters-in-law in Rural China view and negotiate intergenerational contracts with their older adult parents-in-law. A qualitative case study design was used, and multiple data collection methods (including semi-structured interviews, observation and document review) with thematic analysis were employed. Findings highlight that daughters-in-law play a key role in shaping the intergenerational contracts between their spouse and their spouse’s parents. In particular, the daughters-in-law provided instrumental support to their parents-in-law who were without self-care abilities or at risk when their adult child (the daughter-in-law’s spouse) went to the city/county for work. There were also unique findings highlighting diverse negotiations of the intergenerational contract between daughters-in-law and their older adult parents-in-law. In the context of growing austerity and the current pandemic, whereby informal social supports and networks become key to older adults’ wellbeing, identifying strengths and barriers of intergenerational support from daughters-in-law, is significant to support both the individual members and family wellbeing.

